# The newt reprograms mature RPE cells into a unique multipotent state for retinal regeneration

**DOI:** 10.1038/srep06043

**Published:** 2014-08-13

**Authors:** Md. Rafiqul Islam, Kenta Nakamura, Martin Miguel Casco-Robles, Ailidana Kunahong, Wataru Inami, Fubito Toyama, Fumiaki Maruo, Chikafumi Chiba

**Affiliations:** 1 Graduate School of Life and Environmental Sciences, University of Tsukuba, Tennoudai 1-1-1, Tsukuba, Ibaraki 305-8572 Japan; 2 Faculty of Life and Environmental Sciences, University of Tsukuba, Tennoudai 1-1-1, Tsukuba, Ibaraki 305-8572 Japan; 3 Graduate School of Engineering, Utsunomiya University, Yoto 7-1-2, Utsunomiya, Tochigi 321-8585, Japan

## Abstract

The reprogramming of retinal pigment epithelium (RPE) cells in the adult newt immediately after retinal injury is an area of active research for the study of retinal disorders and regeneration. We demonstrate here that unlike embryonic/larval retinal regeneration, adult newt RPE cells are not directly reprogrammed into retinal stem/progenitor cells; instead, they are programmed into a unique state of multipotency that is similar to the early optic vesicle (embryo) but preserves certain adult characteristics. These cells then differentiate into two populations from which the prospective-neural retina and -RPE layers are formed with the correct polarity. Furthermore, our findings provide insight into the similarity between these unique multipotent cells in newts and those implicated in retinal disorders, such as proliferative vitreoretinopathy, in humans. These findings provide a foundation for biomedical approaches that aim to induce retinal self-regeneration for the treatment of RPE-mediated retinal disorders.

During development, the neural retina (NR) and retinal pigment epithelium (RPE) originate from a common cell source, i.e., neuroepithelial cells of the early optic vesicle, and this differentiation is indispensable for proper vision. In the adult stage, the RPE is located between the NR and the choroid and has a highly specialised morphology as well as physiological function^1^,^2^. Mature RPE cells are, as a rule, mitotically quiescent, but when the NR suffers traumatic injury, these cells lose their epithelial characteristics and undergo proliferation and transformation. In humans, this change in RPE cells, categorised as epithelial-mesenchymal transition (EMT), is responsible for retinal disorders, such as proliferative vitreoretinopathy (PVR)^3^. Recently, it was demonstrated that human RPE cells can be reprogrammed into multipotent cells, termed RPE stem cells (RPESCs), which preferably produce mesenchymal cells, such as myofibroblasts, and RPE cells and contribute to PVR^4^. By contrast, in certain urodele amphibians such as the newt, a similar change in RPE cells (termed transdifferentiation) enables regeneration of an entire retina^3^. Thus, newt retinal regeneration can serve as a good model system for comparison with RPE-mediated retinal disorders in humans, and such studies will contribute to the development of medical treatments targeting *in vivo* retinal regeneration.

In the adult newt, when the NR is completely removed from the eye via surgery (i.e., retinectomy), the retina is regenerated from two cell sources^5^,^6^; the primary cell source is the RPE, which regenerates the NR and renews the RPE, and the secondary source is retinal stem/progenitor cells, which are present in the ciliary marginal zone (CMZ) starting at the embryonic stage. These cells extend toward the central retina along the RPE and participate in regeneration of the peripheral portion of the NR. Therefore, by eliminating the peripheral retina, it is possible to focus on retinal regeneration that originates solely from the RPE. We previously described this process using a Japanese fire bellied newt, *Cynops pyrrhogaster*^5^,^6^ (see Supplementary Movie 1). Upon retinectomy, RPE cells are detached from each other as well as from the basement membrane (Bruch's membrane), re-enter the cell cycle, and form cell aggregates [Stage E-1; this event occurs typically between days 5 and 10 post-operation (po). Almost all of the RPE-derived cells at this stage have entered the S-phase of the cell cycle, but they do not proceed into the M-phase. Note that upon retinectomy, the volume of the vitreous cavity decreases and Bruch's membrane, which lies along the RPE, becomes folded because blood vessels in the choroid dilate, allowing increased blood flow as an inflammatory-like response]. The RPE-derived cells generate two rudimentary layers (pro-NR and pro-RPE) for the prospective NR and RPE tissues [Stage E-2; day 14 po; both cell layers at this stage have partially lost melanin pigmentation, and cell division becomes obvious from this stage. Note that both the volume of the vitreous cavity and the shape of Bruch's membrane almost completely recover between Stages E-1 and E-2]. Cells in the pro-RPE layer exit the cell cycle and re-initiate pigmentation, whereas those in the pro-NR layer continue to proliferate [Stage E-3; day 19 po; immunoreactivity (IR) to RPE65 (a marker for terminally differentiated RPE cells) in both layers decreases sharply from this stage]. Thereafter, the RPE layer matures (from approximately day 28 po, RPE65-IR re-appears and microvilli begin to extend) while a new NR is formed (neuronal cell differentiation and network construction are almost complete between days 45 and 65 po).

Thus, in this system, RPE-derived cells preserve pigmentation and RPE65 protein expression until Stage E-3^5^. However, because almost all of the RPE-derived cells at Stage E-1, which seem homogeneous with respect to their pigmentation and RPE65-IR, uniformly express the neural stem cell marker Musashi-1 in the cytoplasm^7^, we hypothesised that adult newt RPE cells are reprogrammed into multipotent cells, which give rise to both a new NR and RPE. We termed these RPE-derived cells ‘stem-like cells’^6^. However, the multipotency of these stem-like cells has not yet been determined.

In this study, we evaluated adult newt RPE-derived stem-like cells using immunohistochemistry and single-cell qPCR and obtained valuable insight into the similarities and differences between retinal disorders and regeneration.

## Results

### Identification of newt Pax6 as a probe for multipotent cells that can generate both the NR and RPE

We predicted that RPE-derived stem-like cells would be comparable to cells capable of differentiating into both the NR and the RPE, such as (i) neuroepithelial cells of the early optic vesicle, (ii) retinal stem/progenitor cells of the pro-NR region in the optic vesicle (late stage)/cup, (iii) cells of the CMZ in embryonic/larval eyes, (iv) cells of the pro-RPE region in the optic vesicle (late stage)/cup, or (v) immature/uncommitted RPE cells in the embryonic/larval eyes, on the basis of evidence from developmental and regeneration biology^8^,^9^,^10^. To test this hypothesis, we selected the transcription factor Pax6 as a probe because this factor is commonly expressed in these cells and its roles are highly conserved in vertebrates^11^. In addition, in studies of embryonic/larval animals, Pax6 has been recognised as a master control gene for the transdifferentiation/fate-switching of RPE cells into NR cells^8^,^12^.

**Figure 1:**
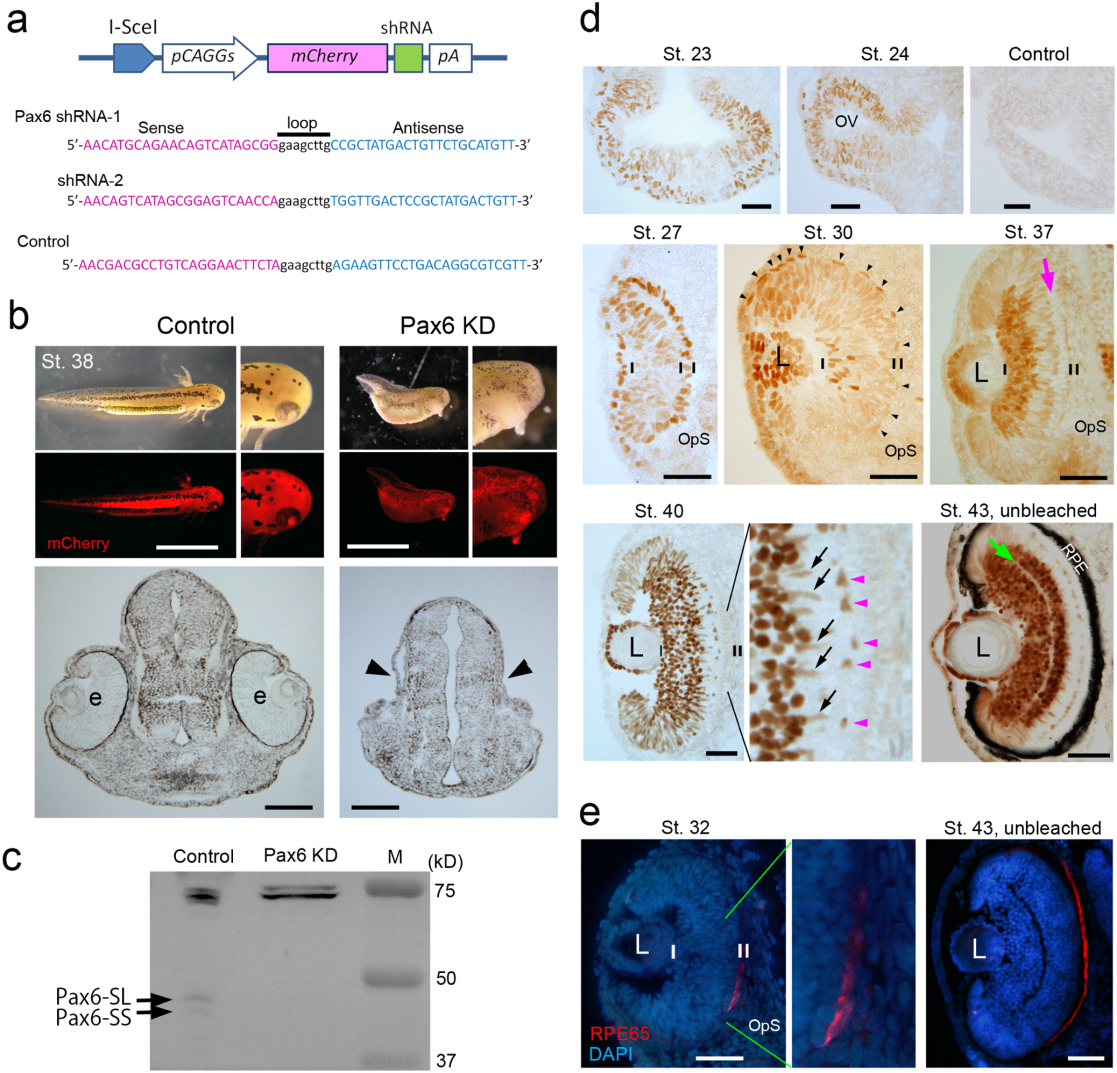
Identification of newt Pax6 as a probe for multipotent cells that can generate both the NR and RPE. (a–c), Functional identification by transgenesis. Knockdown of Pax6 was carried out with two Pax6 shRNAs, which were designed to degrade all four transcript variants (LL, LS, SL, and SS) of *Pax6* (Supplementary Fig. 1a). Each was inserted into a transgene construct, which enables the expression of both a reporter mCherry and *shRNA* in the whole body of the animal (a). Control shRNA was designed from a region in the newt crystallin promoter. Pax6 shRNAs affected eye morphogenesis as well as body growth along the anterior-posterior axis (b). shRNA-2 exerted a more severe effect than shRNA-1 (Supplementary Fig. 1b). In the case of shRNA-2 (Pax6 KD in b), 50% of larvae that showed strong mCherry fluorescence lacked eyes (arrowheads). Western blot demonstrated the knockdown of Pax6 (c). Two bands corresponding to Pax6-SL (~47 kD) and -SS (~45 kD) isoforms were detected in the control lane (Control shRNA; larvae at St. 35–38) but not in the Pax6 KD lane (Pax6 shRNA-2; eye-less larvae at the same age). The bands near 75 kD represent ubiquitous proteins that were stained under the current experimental conditions. (d), (e), Expression patterns of Pax6 (d) and RPE65 (e) during retinal development. Pax6 was expressed, almost uniformly, in both the prospective-NR (pro-NR) and -RPE (pro-RPE) regions in the optic cup (St. 27) as well as in cells in the early (St. 23) and late (St. 24) optic vesicle (*OV*). Pax6 expression in both progenitor layers started to decrease from the central area near the optic stalk (*OpS*) as the eye grew. On the NR side, Pax6 expression increased in certain types of cells as they differentiated in the following order: ganglion/amacrine cells (St. 30) → horizontal cells (St. 37) → Müller glia cells (St. 40). It should be noted that Müller glia cells in this animal expressed Pax6 even in the mature NR (Supplementary Fig. 2). On the RPE side, pigmentation occurred in most areas by St. 28–29. Pax6 expression started to decrease from the central area around St. 30 when the cells there were still proliferating. Proliferation stopped at around St. 32 when the presumptive outer nuclear layer (photoreceptor layer) appeared on the NR side. RPE65, a maturation marker of RPE cells, initiated expression from the corresponding area at St. 32 (red in e). Short vertical lines: borders of NR and RPE; arrowheads in St. 30: nuclei of prospective RPE cells; pink arrow in St. 37: presumptive outer nuclear layer; pink arrowheads and black arrows in St. 40: horizontal cells and Müller glia cells; and green arrow in St. 43: presumptive inner plexiform layer; *L*: lens. Tissues were bleached except for tissues in St. 43. DAPI: a nuclear marker. Scale bars: 5 mm for larvae in b and 100 μm for the other panels.

In the *C. pyrrhogaster* newt, four transcript variants (*Cp-Pax6-LL*, *-LS*, *-SL* and *-SS*) from the same locus have been reported^13^. However, these variants have not been functionally validated, and we were unable to preclude the presence of paralogues because the newt genome (~20 Gbp) has not yet been sequenced. Thus, for the first time, we applied a transgenic protocol that was established for the newt^14^ to knockdown all four Pax6 variants by RNA interference with shRNA, and we confirmed their conserved function in eye morphogenesis. In a severe case with shRNA-2, the larvae demonstrated a small head with no eyes (Fig. 1a–c and Supplementary Fig. 1). Consequently, we concluded that this locus functionally corresponds to *Small eye* (*Sey*) Pax6^15^.

Thus far, the expression patterns of Pax6 during newt eye development and retinal regeneration have been studied by histology^13^,^16^,^17^. However, the cRNA probes or antibodies used in these studies were not able to discriminate canonical paired-homeodomain type Pax6 (including the four Pax6 variants in the newt) from paired-less variants, which should also be expressed in the eye tissues^18^. In addition, background noise in eye tissues that contain early regenerating retinas is too high to discriminate signals^17^. Therefore, we screened an antibody (AD2.38) that is specific to canonical Pax6 (Fig. 1c and Supplementary Fig. 2a), and improved the immunohistochemistry protocols (Methods). Finally, we successfully compared the expression patterns of Pax6 between developing and regenerating retinas.

In embryonic/larval development, as anticipated, the expression patterns of Pax6 were highly conserved (Fig. 1d, e and Supplementary Fig. 2b–d). Pax6-IR was observed in the following: (i) the neuroepithelial cells of the early optic vesicle (St. 23); (ii) the pro-NR region in the optic vesicle (late stage; St. 24)/cup (St. 27); (iii) retinal progenitor cells in immature retinas (St. 30); (vi) the CMZ; (v) the pro-RPE region in the optic vesicle (late stage; St. 24)/cup (St. 27); (vi) immature RPE cells that contained melanin pigments but did not show RPE65-imminoreactivity (St. 30); and (vii) some types of NR cells (ganglion, amacrine, horizontal and Müller glia cells).

### Pax6 immunoreactivity was not detected in RPE-derived stem-like cells but was detected in cells forming the pro-NR layer

**Figure 2:**
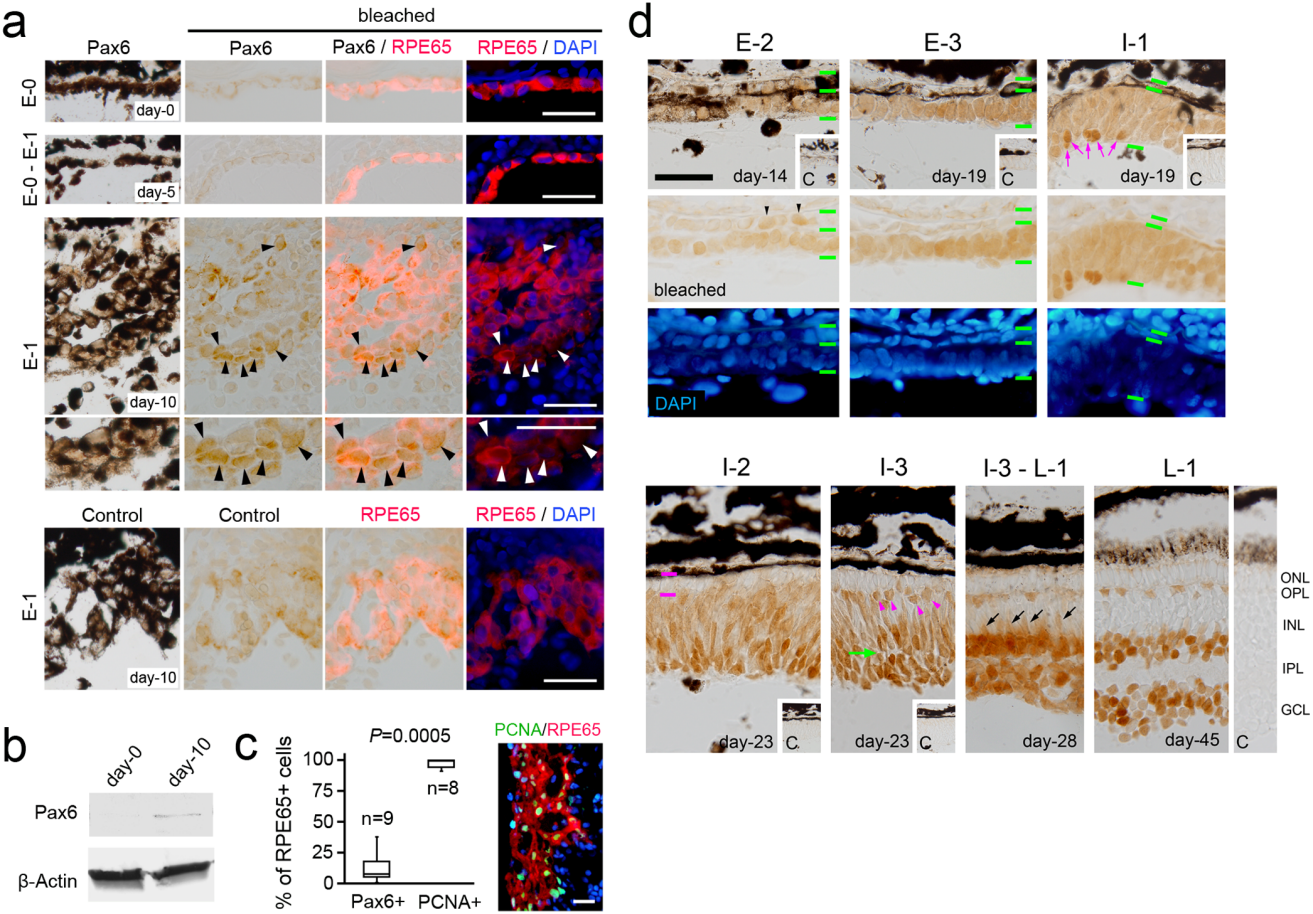
Expression pattern of Pax6 during retinal regeneration. (a–c), Stage E-0 to E-1. RPE cells that were tracked by RPE65-immunofluorescence (red) did not show Pax6-IR (brown) until day 10 after retinectomy (a). In this period, RPE cells lost their epithelial structure to form cell aggregates (St. E-1). Pax6-IR was first detected only in a small number of cells (arrowheads). A Western blot supported the expression of Pax6 at day 10 (b). Protein samples were prepared from RPE-choroid tissues collected from the posterior halves of retinectomised eyeballs on days 0 and 10. The band for day 10 corresponds to the LL isoform (~49 kD). β-Actin: internal control. The cell count in the tissue sections from three different eyes at day 10 demonstrates that almost all RPE-derived cells at this stage had entered the cell-cycle, as revealed by PCNA-IR, but they did not express Pax6 (c). The ratio of Pax6+ cells to RPE65+ cells was significantly lower than that of PCNA+ cells to RPE65+ cells (*P* = 0.0005, n: number of sections; Mann-Whitney U test). (d), Stage E-2 to L-1. When the two rudimentary layers formed (St. E-2) at approximately day 14, Pax6-IR became almost uniformly localised along the inner layer (pro-NR layer), but there were only in a small number of nuclei (arrowheads) along the outer layer (pro-RPE layer). Pax6-IR in the pro-RPE layer became undetectable by day 19 when constituent cells mostly exit the cell-cycle^5^ (St. E-3). By contrast, cells in the pro-NR layer (or the regenerating NR), which continue to proliferate^5^, sustained Pax6-IR. In the ensuing retinal regeneration, the thickness of the regenerating NR increased, and, as cell differentiation proceeded, the number of progenitor cells decreased, while Pax6-IR NR cells appeared in the following order: ganglion/amacrine cells (pink arrows, St. I-1) → horizontal cells (pink arrowheads, St. I-3) → Müller glia cells (arrows, St. I-3 to L-1). Thus, after the two rudimentary layers had formed, the process of retinal regeneration was similar to that of embryonic/larval retinal development. Green horizontal lines in St. E2 to I-1: borders of NR and RPE; pink horizontal lines in St. I-2: width of the presumptive outer nuclear layer (*ONL*); green arrow in St. I-3: presumptive inner plexiform layer (*IPL*); panels labelled with ‘c’: negative control; *OPL*: outer plexiform layer; *INL*: inner nuclear layer; *GCL*: ganglion cell layer. Scale bars: 40 μm for a and c and 100 μm for d.

In adult retinal regeneration, if RPE-derived stem-like cells are comparable to cells in the optic vesicles/cups or to immature RPE cells, they should express Pax6. However, unexpectedly, Pax6-IR was first detected in only a small number of RPE-derived cells (mean: 11.8%, n = 9) at Stage E-1 (day 10 po) when almost all of the cells (mean: 97.2%, n = 8) had entered the cell cycle (Fig. 2a–c). This became obvious in subsequent days when RPE-derived cells were segregated into two rudimentary layers (pro-NR and pro-RPE). At Stage E-2 (day 14 po), Pax6-IR was almost uniformly observed along the pro-NR layer, but its expression was very sparse in cells located along the pro-RPE layer (Fig. 2d). Then, Pax6-IR in the pro-RPE layer became undetectable by Stage E-3 (day 19 po, Fig. 2d). By contrast, the thickness of the Pax6-IR+ pro-NR layer or regenerating NR increased, and as cell differentiation proceeded, the number of Pax6-IR+ retinal progenitor cells decreased, while the Pax6-IR+ NR cells appeared in the following order: ganglion cells (St. I-1) → amacrine cells (St. I-2) → horizontal cells (St. I-3) → Müller glia cells (St. I-3 to L-1). Thus, together with our previous findings^5^,^6^, after Stage E-3, the process of retinal regeneration seemed to recapitulate embryonic retinal development.

Consequently, the RPE-derived cells at Stage E-1 were most likely not comparable to the cells we had predicted. In addition, two cell populations seemed to appear abruptly in the RPE-derived cells between Stages E-1 and E-2, forming the pro-NR layer (Pax6-IR+) and the pro-RPE layer (Pax6-IR-) by Stage E-2.

### Single-cell qPCR revealed that adult newt RPE cells express mutipotent properties while preserving their original characteristics

Next, we investigated whether RPE-derived cells express other stem cell markers. To do so, a single-cell qPCR technique was adopted because there are few antibodies available for this model animal, and *in situ* hybridisation seems empirically to have insufficient sensitivity (or signal to noise ratio) for detecting low concentrations of mRNAs whose transcription has started. Candidate genes were identified using a newt *de novo* assembly transcriptome database (IMORI, http://antler.is.utsunomiya-u.ac.jp/imori/), which we concurrently established for the study of early processes of retinal regeneration (Nakamura, K. et al., unpublished). Here, we examined three pluripotency factors (c-Myc, Klf4 and Sox2)^19^, the microphthalmia factor Mitf^20^, and Pax6. Recent *in vitro* studies in mammals have suggested that a set of pluripotency factors can induce the cell type switching of RPE cells into multipotent cells^21^. However, we must note that the expression of Oct4 or Nanog, pluripotency factors that can generate induced pluripotent stem (iPS) cells in mammals^19^,^21^, was not detected in the early processes of retinal regeneration (Nakamura, K. et al., unpublished). In other vertebrates, Mitf is known to be expressed in (i) the neuroepithelial cells of the early optic vesicle, (ii) cells of the pro-RPE region in the optic vesicle (late stage)/cup, and (iii) immature/uncommitted RPE cells in embryonic/larval eyes (Supplementary Table 1). In those cells, it has been suggested that loss of function of Mitf is sufficient to induce their fate-switching into the NR^20^.

**Figure 3:**
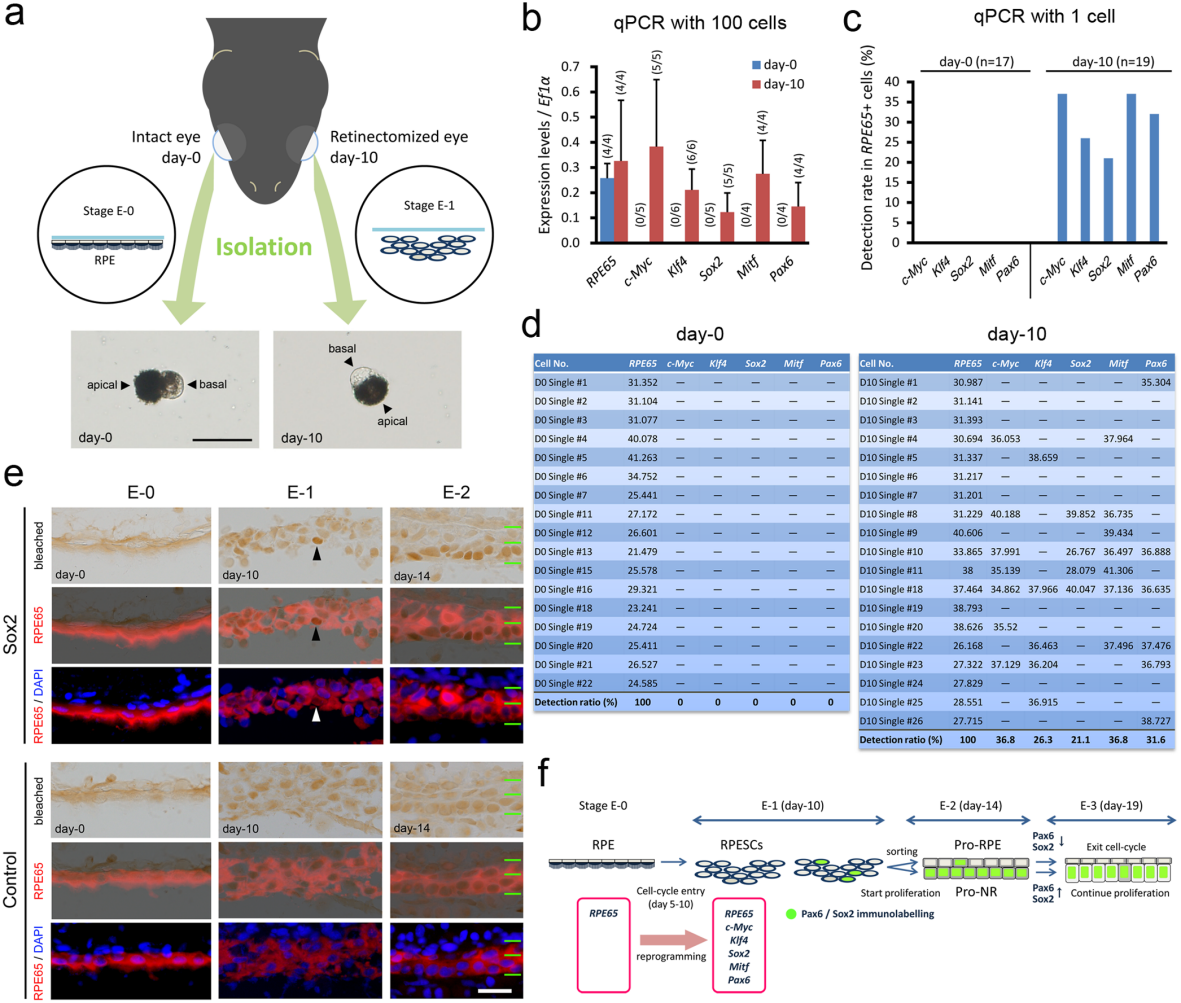
Adult newt RPE cells newly express multipotent properties upon retinectomy while preserving their original characteristics. (a–d), Single-cell qPCR. Normal RPE cells (day 0) and RPE-derived cells (day 10) were isolated from the control and retinectomised eyes of the same animal at day 10 after retinectomy (a). In each-day sample, RPE cells were identified by their morphological characteristics (cells with a pigmented apical part and a non-pigmented part with the nucleus), and solitary cells were manually selected under a dissecting microscope. First, 100 cells on days 0 and 10 were harvested into different tubes to prepare each cDNA sample, and real-time PCR was carried out using these samples for *RPE65*, *c-Myc*, *Klf4*, *Sox2*, *Mitf*, *Pax6* and *Ef1α*. A comparison of the expression levels of genes that compensated for *Ef1α* is shown in b. Numbers in parentheses represent the ratio of detection (dominator: sample number). The same experiments were performed with one-cell samples. The cumulative detection rates of genes in *RPE65*+ cells are shown in c. Raw data tables mentioning the Cq values are shown in d. Horizontal bars indicate no detection. e, Sox2 immunohistochemistry in Stage E-0 to E-2 regenerating retinas. Tissues were bleached. Sox2-IR was first detected in a small number of RPE-derived cells at stage E-1 (arrowhead) and then along the inner rudimentary layer (pro-NR layer) at stage E-2. Green horizontal lines in stage E-2: borders of the pro-NR and pro-RPE layers. Scale bars: 50 μm for a and e. f, Summary of the current results and proposed mechanism.

We isolated RPE cells (day 0 sample) and RPE-derived cells (day 10 sample) from intact and retinectomised eyeballs, respectively, which were collected from animals at day 10 po (Fig. 3a). Then, we selected solitary cells manually under a dissecting microscope, followed by cDNA synthesis and qPCR. For the day 0 sample, RPE cells were identifiable by their characteristic morphology (i.e., polarity with a pigmented apical region and a non-pigmented basal region containing the nucleus). However, in the day 10 sample, visual cell identification was difficult because of potential contamination of pigmented cells of different origins^5^. Therefore, only cells that resembled normal RPE cells were selected, i.e., a fraction of the RPE-derived cells whose morphological change was slightly delayed.

We first carried out qPCR analysis using 100 harvested cells (Fig. 3b). Pluripotency factors (*c-Myc*, *Klf4* and *Sox2*), *Mitf* and *Pax6* were consistently detected in the day 10 samples but never in the day 0 samples (n ≥ 4). Although *RPE65* was detected in both samples (n = 4), the relative expression levels of *RPE65* were not significantly different between the samples. We next performed qPCR with single-cell samples (Fig. 3c, d). On day 0, as observed from 100 single-cell samples, none of the genes examined were detected. However, in a total of 17 cells, *RPE65* was detected (*RPE65+* cells). By contrast, on day 10, from a total of 19 *RPE65+* cells, 13 cells (68%) showed the expression of target genes (Fig. 3c), although the combination of detected genes varied between individual cells (day 10 in Fig. 3d).

Such a fluctuation in the detection of each target gene in the day 10 samples may be explained by the probability of reverse transcription (RT) under a condition of low concentrations of mRNA (the substrate of the RT polymerase), which should be true for the current single-cell samples. That is, the detection rate should depend on the concentration of mRNA under this condition. As a result, all data were combined (n = 19), and the detection rates of target genes (Fig. 3c) showed a similar pattern to that (*c-Myc* > *Klf4* > *Sox2*; *Mitf* > *Pax6*) of their relative expression levels obtained in the 100 single-cell samples (Fig. 3d). These observations suggest that mRNAs of all the target genes may be present in a single day 10 cell at different concentrations. It is noteworthy that one particular cell (D10 Single #18) expressed all target genes (Fig. 3d). Taken together, these findings indicate that RPE cells are reprogrammed into a unique multipotent state.

To visualise the expression of these multipotency factors, immunohistochemistry was attempted. We searched for antibodies for c-Myc, Klf4, Sox2 and Mitf in this species, and we finally found a good antibody for Sox2 (Supplementary Fig. 3a). In both the developing and adult eyes, the patterns of Sox2-IR were consistent with those reported in other vertebrates^22^,^23^,^24^; Sox2-IR was observed in (i) the neuroepithelial cells of the early optic vesicle (St. 23), (ii) the pro-NR region in the optic vesicle (late stage; St. 24)/cup (St. 27), (iii) retinal progenitor cells in the immature retina (St. 29, 30, 32), (vi) the CMZ, and (v) some types of NR cells (amacrine and Müller glia cells) (Supplementary Fig. 3b–d and Supplementary Table 1).

Unexpectedly, but interestingly, during adult retinal regeneration, the patterns of Sox2-IR expression until Stage E-3 were almost the same as those for Pax6 (Fig. 3e). Sox2-IR became detectable in a small number of RPE-derived cells at Stage E-1 (day 10 po) and was clearly observed along the pro-NR layer at Stage E-2 (day 14 po).

## Discussion

In conclusion, the results of our experiments are summarised and explained in Figure 3f. In the adult newt, the RPE is the sole cellular source for regeneration of missing NR in the posterior eye. The adult newt RPE is a highly specialised monolayer, similar to that in other vertebrates^5^. We can exclude the possibility that the intact RPE contains ‘retinal stem/progenitor cells resembling RPE cells (or RPE65-IR+)’ that function to regenerate the NR because RPE-mediated retinal regeneration after retinectomy could be repeated more than three times (the cellular source for the new NR was not exhausted; our unpublished observation) and the RPE was not replaced by tissues other than the RPE itself^5^. Moreover, the current single-cell qPCR results revealed that intact RPE cells do not express retinal stem/progenitor markers.

Upon retinectomy, RPE cells are detached from each other as well as the basement membrane, lose their epithelial characteristics to become cell aggregates, and enter the S-phase of the cell cycle. These RPE-derived cells are segregated into two rudimentary layers (the pro-NR and pro-RPE layers) with correct polarity, eventually regenerating a new NR while renewing the RPE. Our previous study demonstrated that almost all of the RPE-derived cells that entered the S-phase of the cell-cycle on day 10 po (Stage E-1) uniformly expressed the neural stem-cell marker Musashi-1 in the cytoplasm, and the expression of Musashi-1 was sustained along the pro-NR layer at day 14 po (Stage E-2) but was down-regulated along the pro-RPE layer^7^. These observations led us to hypothesise that RPE cells are reprogrammed into multipotent cells by Stage E-1 and are then differentiated into two cell populations, which may depend on their surrounding microenvironment (or niche), between Stages E-1 and E-2.

In this study, we found that RPE cells newly express *c-Myc*, *Klf4*, *Sox2*, *Mitf* and *Pax6* while retaining their expression of RPE65, as observed for both the mRNA and protein levels. These cells are thus converted into a unique state of multipotent cells (termed RPESCs here for convenience; Figure 3f). Moreover, the pluripotency factors c-Myc, Klf4 and Sox2 may reprogram or initialise RPE cells, as demonstrated in *in vitro* studies in mammals^21^, whereas Oct4 and Nanog are unlikely to participate in this system, as observed in chick RPE reprogramming into the NR^10^ as well as in other regeneration systems in adult newts^25^. Our review of the gene expression patterns during eye morphogenesis (Supplementary Table 1) suggests that the cells that express c-Myc, Klf4 and Sox2, as well as Pax6 and Mitf, are only those in the early optic vesicle. Hence, the adult newt RPESCs seem to correspond to the neuroepithelial cells of the early optic vesicle, which generate both the NR and RPE. In other words, adult newt RPE cells seem to dedifferentiate in nature. Our previous study suggested the presence of RPE65 expression and melanin pigments in RPE-derived cells for a longer period (~20 days after retinectomy), which may be due to their slow degradation or excretion^5^.

However, both the Pax6 and Sox2 antibodies, which clearly labelled optic vesicle cells, did not label RPESCs. In the tissue of Stage E-1 (day 10 po), although single-cell qPCR detected *Pax6* and *Sox2* mRNA expression even in RPE-derived cells that had not reached a standard progression with a cell shape resembling that of a normal RPE cell, the antibodies labelled only the advanced cells that formed the pro-NR layer. Thus, perhaps either the level of protein expression in RPESCs is very low or some type of modification occurs to both proteins to prevent the antibodies from accessing the corresponding epitopes. However, the latter explanation can be excluded because we detected Pax6 protein expression by Western blotting (Fig. 2b). If the former explanation is true, at least with respect to Pax6 and Sox2, adult newt RPESCs may not require expression levels equivalent to those in optic vesicle cells to behave as multipotent cells.

Establishing a primary pattern of tissue organisation at an early stage of regeneration is essential for the reconstruction of different tissue types, with the correct polarity, from a single cell source. Pax6/Sox2-IR+ cells appeared among RPESCs from Stage E-1 to E-2, which formed the pro-NR layer (Fig. 3f). By contrast, the cells with no immunoreactivity formed the pro-RPE layer. Because mitotic figures were clearly observed in RPE-derived cells between Stages E-1 and E-2, these cell populations seemed to be arranged according to the correct polarity (inside: cells forming the pro-NR; outside: cells forming the pro-RPE), and their proliferation was activated during this period.

However, this process was imperfect, as a small number of Pax6/Sox2-IR+ cells were irregularly distributed in the pro-RPE layer (termed ‘displaced pro-NR cells’ here). Interestingly, because the displaced pro-NR cells participated in the renewal of the RPE, RPESCs directed towards regeneration of the NR seem to retain the ability to re-differentiate into the original cell type. Pax6/Sox2-IR in displaced pro-NR cells disappeared between Stages E-2 and E-3, whereas along the pro-NR layer, Pax6/Sox2-IR seemed to be sustained until Stage I-1 when neuronal cell differentiation began, suggesting that opposite regulation of Pax6/Sox2 expression promotes the renewal of the RPE and regeneration of the NR.

Considering their morphological and physiological changes^5^,^6^, the pro-NR and pro-RPE layers seem to be comparable to the pro-NR and pro-RPE regions in the optic vesicle (late stage)/cup. However, unlike the pro-RPE region (Pax6-IR+/Sox2-IR-) in the optic vesicle/cup, the pro-RPE layer (Sox2-IR-) was seldom labelled with the Pax6 antibody. During retinal development, Pax6-IR seemed to be regulated in association with cell differentiation. As for the RPE, Pax6-IR observed in immature cells disappeared as cells matured while expressing RPE65 (Fig. 1d, e). In retinal regeneration, RPE65-IR reappeared along the renewing RPE between Stages I-3 and L-1 (day 28 po)^5^. Thus, compared to the developmental process, the disappearance of Pax6-IR in the renewing RPE seems to occur far earlier than the maturation of the RPE, implying that there are different regulatory mechanisms for Pax6 between development and regeneration. As discussed for RPESCs, the process of RPE renewal might not require Pax6 at levels equivalent to those for development of the RPE. Interestingly, a published report indicated that another transcription factor, Otx2, which is required for RPE cell differentiation during eye development, might be involved in the maintenance and specification of RPE cells during retinal regeneration^26^.

Taken together, to the best of our knowledge, this study provides the first set of evidence at the single cell level for the natural reprogramming of somatic cells for the regeneration of body parts in the newt. Furthermore, our results reveal that adult newt retinal regeneration has many important issues that require further investigation, such as reprogramming, multipotency, cell specification, niche, and tissue patterning as well as EMT and proliferation. However, to apply this knowledge to medicine, the extent to which this system is unique to this animal must be evaluated. In humans, upon retinal injury, RPE cells lose their epithelial characteristics and acquire the ability to migrate and proliferate, transforming into mesenchymal cells such as myofibroblasts^3^,^4^. In PVR, the RPE-derived myofibroblasts are a major component of the epiretinal membrane that covers the wound of the NR, although the membrane eventually contracts together with the NR, leading to loss of vision. Thus, the initial response of human RPE cells to retinal injury resembles that of adult newt RPE cells. However, this phenomenon is categorised as EMT because the fate of the RPE cells is mesenchymal tissue rather than NR^3^. Recent studies in stem cell biology have reported that human RPE cells pass through a multipotent state during EMT, and *in vitro* studies suggest that RPE-derived proliferative cells in humans are capable of expressing C-MYC and KLF4 as well as PAX6 and MITF (but not OCT4 or NANOG) and behave as stem cells (termed RPESCs)^4^. Interestingly, in human RPESCs, SOX2 expression seems to be negligible^4^, which is different from that observed in adult newt RPESCs. Because Sox2 is considered a decisive factor for neural competence in the retina^23^,^24^, this factor, and possibly its co-factors, may be important for directing RPESCs toward retinal regeneration. This study revealed that human and newt RPESCs show close similarities in their intrinsic properties. In the future, comparative/integrated studies of these regeneration-competent and -incompetent models at the molecular level, such as through comprehensive single-cell transcriptome analysis, would likely provide essential factors for inducing retinal regeneration in human RPESCs as well as understanding the molecular mechanisms of adult newt retinal regeneration.

## Methods

All experiments were carried out in accordance with the guidelines approved by the University of Tsukuba Animal Use and Care Committee (AUCC).

### Animals

The Japanese fire bellied newt, *Cynops pyrrhogaster*, was used for this study. Fertilised eggs for transgenic and developmental studies were obtained from the adult Toride-Imori, a race of this species that was originally captured from Kamogawa City, Chiba Prefecture, Japan, and were stocked/cultured in the laboratory (Univ. of Tsukuba) and at ‘Imori-no-Sato’ (Toride City, Ibaraki Prefecture, Japan; http://imori-net.org/)^14^. In brief, before the experiments, males and females (total body length: male, ~9 cm; female, 11–12 cm) were transferred into a two-aquarium-tank (TAT) system and reared under a semi-natural condition, allowing them to display courtship behaviour, which was followed by the delivery of a spermatophore from the male to the female. To collect fertilised eggs, female newts were injected subcutaneously in the abdominal region with 50 μl (30 U) of gonadotropin (Gonatropin 3000; Asuka Pharmaceutical, Tokyo, Japan) every other day. Wild-type embryos and larvae were reared as described previously^14^. The developmental stage was determined according to previous criteria^14^.

For the regeneration study, adult newts of other races (captured from Kagawa, Okayama, or Miyagi Prefecture) that were purchased from a supplier (Aqua Grace, Yokohama, Japan) were also used. These newts were reared in containers at ~18°C under natural light until the experiments were performed. They were fed daily with frozen mosquito larvae (Akamushi; Kyorin, Himegi, Japan), and the containers were kept clean at all times^14^.

### Transgenesis

An *I-SceI* transgenic protocol for the newt^14^ was applied to knockdown Pax6. A transgene construct pCAGGs-mCherry-shRNA (Sce) (Fig. 1a) was prepared by modifying pCAGGs-EGFP (Sce)^14^ with conventional molecular cloning techniques; a reporter EGFP was replaced with mCherry to minimise autofluorescence, and then a sequence encoding *shRNA* was inserted, with restriction enzymes *Mun I* (5′ end) and *Hpa I* (3′ end), in between the mCherry and the polyA signal. Two shRNAs were designed to degrade all four spliced variants of *Pax6* (Supplementary Fig. 1). A control shRNA was designed using a region (319–342) in the newt crystallin promoter (DDBJ/GenBank Accession #: AB113881). Preparation of single-cell embryos, microinjection of the construct [injection volume: 10 µl/embryo; contents: 200 ng of the DNA construct, 0.5 U of *I-SceI* (R0694S; New England Biolabs Japan, Tokyo, Japan), 1x *I-SceI* buffer, and 0.01% phenol red], and rearing of the injected animals were performed according to previously published protocols^14^.

### Surgical operations

Surgical removal of the NR (retinectomy) was performed as described previously^5^. In brief, after the adult newts were anesthetised in a 0.1% FA100 (4-allyl-2-methoxyphenol; DS Pharma Animal Health, Osaka, Japan) solution in the dark at room temperature (RT, ~22°C) for 2 h, the dorsal half of the left eye was cut open along the position that was slightly below the boundary between the cornea and sclera, and the NR was carefully removed together with the lens. At this time, the retinal margin containing the *ora serrata* (the tissue harbouring the retinal stem/progenitor cells) was also removed. After the operation, the eye flap, consisting of the iris and cornea, was carefully placed back in its original position. The operated animals were allowed to recover in a moist container and were then reared in an incubator (~22°C; 12 h:12 h day:night cycle) until use. The stage of retinal regeneration and corresponding day po were determined according to previous criteria^5^ (Supplementary Movie 1).

### Collection of eyeballs and preparation of tissue samples

Newts were sacrificed under anaesthesia to minimise suffering^5^. To collect eyeballs, the animals were decapitated, and the eyeballs were carefully enucleated with fine scissors and forceps. In some experiments, the eyeballs were first dissected into the eye cups (posterior half of the eyeball), and then the NR (in the case of normal eyeballs) or the RPE-choroid tissues were collected^17^. For embryos/larvae, the whole body served as a tissue sample.

### Antibodies

The primary and secondary antibodies used for immunoblotting and immunohistochemistry were as follows: for Pax6, mouse monoclonal anti-Pax6 antibody (1:100; AD2.38, sc-32766; Santa Cruz Biotechnology, TX 75220, U.S.A) and biotinylated goat anti-mouse IgG antibody (1:500; Vector Laboratories, CA 94010, U.S.A); for Sox2, rabbit anti-Sox2 antibody (1:660; ab97959; Abcam, Cambridge, U.K.) and biotinylated goat anti-rabbit IgG antibody (1:500; Vector Laboratories); for RPE65, mouse monoclonal anti-RPE65 antibody (1:500; MAB5428; Millipore, Darmstadt, Germany) and tetramethylrhodamine-conjugated goat anti-mouse IgG antibody (1:200; T2762; Life Technologies, MD 20850, U.S.A); and for PCNA, human autoantibody to PCNA (1:500; gift from Dr. T. Saito)^5^ and Alexa Fluor 488-conjugated goat anti-human IgG (1:1,000; A-11013; Life Technologies). For the negative control, mouse or rabbit antibodies (IgG) with no specific reactivity to newt tissues (*e.g*., anti-dsRed antibody; 632543 or 632496; Clontech, CA 94043, U.S.A) were applied, instead of the primary antibody, at the same concentration of IgG.

### Immunoblotting

Protein sample preparation and immunoblotting were performed as described previously^5^,^17^. In brief, tissue samples (*i.e*., embryos/larvae or eye tissues) were collected in chilled PBS and dissolved in lysis buffer [25 mM Tris, 150 mM NaCl, 1 mM EDTA·2 Na, 1% Igepal CA-630, 1% proteinase inhibitor cocktail (P-8340, Sigma, MO 63103, U.S.A), 1% sodium deoxycholate, and 0.1% SDS, pH 7.5] at a concentration of one tissue sample/7 µl lysis buffer. The supernatant was mixed with an equal volume of 2× sample buffer [100 mM Tris-HCl (pH 6.8), 4% SDS, 20% glycerol, 0.01% (w/v) bromophenol blue (Wako, Osaka, Japan), and 10% β-mercaptoethanol (Sigma)], boiled for 5 min, and then stored at −20°C until use. Proteins were separated on a 10% gel (456–1033; Mini-Protean TGX Gels; Bio-Rad, CA 94547, U.S.A) by SDS-PAGE and transferred onto a PVDF membrane (Immun-Blot PVDF membrane for protein blotting, Bio-Rad). The blots were conventionally labelled with the primary antibody and visualised with the biotinylated secondary antibody and an ABC/DAB system (Vectastain ABC Elite kit, DAB substrate kit, Vector Laboratories).

### Immunohistochemistry

Tissue samples were fixed in 2% paraformaldehyde/0.2% picric acid in PBS (pH 7.4) at 4°C for different periods [embryos/larvae, 8 h; normal and retinectomised (day 5 to 45 po) eyeballs, 5 h; eyeballs immediately after retinectomy (*i.e*., day 0 po), 2 h] and then cryosectioned transversely at a thickness of ~20 µm^5^.

Immunolabelling of tissue sections was performed as described previously^5^ with some modifications. For eyeball sections, an antigen retrieval step was added before the process of immunolabelling; after the slits were made along the inside margin of the cornea and sclera to separate the iris and retinal tissues from those connective tissues (by manipulating a blade under a dissecting microscope), eyeball sections were rinsed in PBS for 15 min, incubated in a sodium citrate buffer (10 mM sodium citrate, 0.05% Tween 20, pH 6.0) at 90°C for 10 min, and then rinsed in PBS twice for 5 min each. During incubation in the sodium citrate buffer, the corneal and scleral tissues became coiled and dissolved into the buffer solution. This treatment clearly decreased the background staining of the tissues (possibly due to some blood-related content in the choroid) while sustaining immunoreactivity, which increased the signal to noise ratio.

The localisation of Pax6 and Sox2 was determined using a previously developed immunoperoxidase labelling procedure with the ABC/DAB system^5^, and RPE65 and PCNA were evaluated with an immunofluorescence procedure^5^. In the case of double labelling with Pax6 and RPE65 antibodies (both are mouse monoclonal antibodies), immunoperoxidase labelling of Pax6 without the DAB reaction was followed by immunofluorescence labelling of RPE65. Finally, Pax6-IR was visualised with DAB. In some experiments, the melanin pigments of cells were bleached to reveal the immunoreactivity^5^. After labelling, cell nuclei were counterstained with DAPI (1:50,000; D1306; Life Technologies).

### Cell isolation and cDNA synthesis

For single-cell qPCR, both the right (intact) and left (retinectomised) eyeballs of animals at day 10 po were used (Fig. 3a). In particular, the right eyeballs were used for the day 0 po sample containing intact RPE (stage E-0), and the left eyeballs were used for the day 10 po (stage E-1) samples.

For one round of the experiment, three animals were sacrificed under anaesthesia, and the right and left eyeballs were collected in different plastic dishes (Falcon 35-3001; Becton Dickinson, NJ07417, U.S.A) that were filled with RNase-free PBS on ice. The right eyeball (day 0) was dissected into the eye cup, the NR was removed, and then the RPE sheet together with the choroid tissues was isolated by separating these from the sclera using a fine pin and forceps (left-hand pathway in Fig. 3a)^17^. Subsequently, the left eyeball (day 10) was carefully opened from the wound at the time of retinectomy using a fine pin and scissors, the anterior part of the eyeball containing the iris and ciliary marginal zone was carefully removed, and RPE-derived cells in the posterior eye were collected together with the choroid tissues in the same manner as for the right eyeball (right-hand pathway in Fig. 3a). After as many blood cells in the choroid were removed as possible by shaking the samples in the dish, three samples for each day were transferred into different 15-ml tubes containing 1 ml of elastase (1 mg/ml; Porcine pancreas; Ref: 11027891001; Roche Diagnostics Japan, Tokyo, Japan) in EDTA solution (in mM: 115 NaCl, 3.7 KCl, 10 EGTA, 18 D-glucose, 10 HEPES, and 0.001% phenol red, pH 7.5 adjusted with 0.3N NaOH) with a 3.5 ml transfer pipette (Sarsted, D-51588 Nümbrecht, Germany). The samples were then incubated for 90 min at 28°C. The tissue samples were rinsed with a chilled newt saline solution (in mM: NaCl, 115; KCl, 3.7; CaCl_2_, 3; MgCl_2_, 1; D-glucose, 18; and HEPES, 5; pH 7.5 adjusted with 0.3N NaOH) several times and then dissociated by gentle trituration with the transfer pipette. The cell suspension was transferred into a 1% agarose-coated 35-mm plastic dish (Falcon 35-3001) that was placed on ice. These trituration and cell collection steps were repeated 5–6 times.

cDNA synthesis and pre-amplification were carried out, under conventional nuclease-free conditions, using TaKaRa CellAmp Whole Transcriptome Amplification Kits (Real Time) Ver.2 (code#: 3730; Takara, Otsu, Japan) according to the manufacturer's instructions. In brief, solitary RPE cells or RPE-derived cells, which were identified by their morphological characteristics (Fig. 3a), were picked up from the dish using a microtip (703Y, Ina Optika, Osaka, Japan) attached to a micropipette (set at 0.5 µl; Pipetman P20; Gilson, WI 53562-0027, U.S.A.) under a dissecting microscope. For 100-cell qPCR analysis, 100 cells were transferred into a PCR tube (No T-02F, Ina Optica) containing 50 µl of RNase-free PBS, and the cells were settled on the bottom of the tube with centrifugation for 1 min at 3,000 g. After the PBS in the tube was discarded, leaving behind ~0.5 µl, a 4.5-µl reaction mixture containing cell lysis buffer was added. For one-cell qPCR analysis, one cell (0.5 µl) was directly transferred into the tube containing the 4.5-µl reaction mixture. In both samples, cDNA synthesis was followed, and then the resulting cDNAs were amplified non-selectively by PCR (95°C, 1 min → 50°C, 1 min → 72°C, 3 min: 1 cycle; 95°C, 30 sec → 67°C, 1 min → 72°C, 3 min: 20 cycles; 72°C, 1 min). The amplified cDNA samples were stored at −20°C until use.

### Quantitative PCR (qPCR)

The amplified cDNA samples were diluted 10x and then used as a template for qPCR. qPCR was performed using a LightCycler® Nano system (Roche Applied Science, Penzberg, Germany) according to the manufacturer's instructions for the FastStart Essential DNA Green Master (Roche) or FastStart Essential DNA Probes Master (Roche) for 45 cycles. For each gene examined in this study, the DDBJ/GenBank accession number, primers, probes [selected from the Roche Universal Probe Library (https://www.roche-applied-science.com)], and expected size were as follows: *Ef1α*, AB00558, forward: cgtgacatgaggcagactgt, reverse: tagaggccttctgggctgat, 100 bp; *RPE65*, AB095018, forward: tgctgctggaaaggatttga, reverse: gctttctctgcatttctcttcac, probe#: 48, 95 bp; *c-Myc*, AB904156, forward: ctcagaagaagaacaggatgacg, reverse: atccaggcttcctgccagtag, 101 bp; *Klf4*, AB904164, forward: ggtcaagggctggttagtcc, reverse: tgaggaggacacttgatggc, 101 bp; *Sox2*, AB074258, forward: gtttatggaggagggcacgg, reverse: acctgagggacatgatcagc, 119 bp; *Mitf*, AB904165, forward: cgtagcaagatccgtgatgtc, reverse: cagcagttcatccgagcat, probe#: 25, 78 bp; and *Pax6*, D88741, forward: tctgggcaggtattacgagac, reverse: cgatcttgctcaccacctc, probe#: 58, 95 bp. The specificity of the PCR results was confirmed by standard gel electrophoresis and DNA sequencing.

### Data analysis

In the transgenic analysis, bright-light and fluorescence images of embryos/larvae were acquired using a digital camera (C-5060; Olympus, Tokyo, Japan) attached to a fluorescence stereomicroscope (Leica M165 FC; Leica Microsystems, Wetzlar, Germany) with a filter set for mCherry (Exciter: XF1044, 575DF25; Emitter: XF3402, 645OM75; Opto Science, Tokyo, Japan). Embryos/larvae with visible red fluorescence throughout the body were selected and then subdivided into ‘weak’, ‘moderate’ and ‘strong’ on the basis of their fluorescence intensity. The fluorescence intensity was estimated as the average luminance by analysing the fluorescence images of the embryos/larvae that were acquired with a x40 objective lens, using a function of Photoshop Extended CS5 graphics software (Adobe Systems, CA 95110-2704, U.S.A.). Embryos/larvae with an average luminance value of 40–60 were categorised as ‘moderate’, and those with higher and lower values were categorised as ‘strong’ and ‘weak’, respectively.

For the immunohistochemical analysis, bright light and fluorescence images of tissue sections were acquired using a CCD camera system (DP73; cellSens Standard 1.6; Olympus) attached to a fluorescence microscope (BX50; Olympus).

In single-cell qPCR analysis, 100-cell qPCR for each gene, which was always run simultaneously with day 0 and day 10 po samples, was repeated using more than four sets of independently collected samples. The expression levels of each gene (*i.e*., the levels of transcripts estimated from the Cq value) were compensated for *Ef1α* in the same sample. For one-cell qPCR, samples in which *RPE65* was detected were used for further analysis of gene expression.

Data are presented as a box plot graph (Fig. 2b) or as a bar graph showing the mean ± SEM (Fig. 3b). Significant differences were evaluated with the Mann-Whitney's U test or Sheffe's test following the Friedman test. Figures were prepared using Photoshop Extended CS5 (Adobe Systems). Image, brightness, contrast, and sharpness were adjusted according to the journal's guidelines.

## Author Contributions

M.R.I. and C.C. designed and performed all experiments and co-wrote the paper. K.N. and A.K. contributed to the single-cell qPCR analysis and data confirmation by Sanger's DNA sequencing. M.M.C.-R. and A.K. contributed to the transgenic analysis. F.M. maintained Imori-no-Sato together with residents and supplied Toride-Imori. W.I. contributed to the evaluation of antibodies, immunohistochemistry, and Western blotting. F.T. contributed to searching genes with a *de novo* assembly transcriptome.

## Supplementary Material

Supplementary Information Supplementary Movie 1

Supplementary Information Supplementary information
