# Supplementary material for: The newt reprograms mature RPE cells into a unique multipotent state for retinal regeneration
Source: Sci Rep. 2014 Aug 13;4:6043. doi: 10.1038/srep06043 (PMC4131214; doi:10.1038/srep06043)
Supplement: Supplementary Information [file srep06043-s2.pdf]

Supplementary Information

The newt reprograms mature RPE cells into a unique multipotent state for retinal regeneration

Md. Rafiqul Islam[1], Kenta Nakamura[2], Martin Miguel Casco-Robles[1], Ailidana Kunahong[1], Wataru Inami[1], Fubito Toyama[3], Fumiaki Maruo[2] and Chikafumi Chiba[2*]

[1]Graduate School of Life and Environmental Sciences, University of Tsukuba, Tennoudai 1-1-1, Tsukuba, Ibaraki 305-8572 Japan

[2]Faculty of Life and Environmental Sciences, University of Tsukuba, Tennoudai 1-1-1, Tsukuba, Ibaraki 305-8572 Japan

[3]Graduate School of Engineering, Utsunomiya University, Yoto 7-1-2, Utsunomiya, Tochigi 321-8585, Japan

***Correspondence to:** Chikafumi Chiba, Faculty of Life and Environmental Sciences, University of Tsukuba, Tennoudai 1-1-1, Tsukuba, Ibaraki 305-8572 Japan.

Tel: +81-29-853-4667; Fax: +81-29-853-6614; E-mail: chichiba@biol.tsukuba.ac.jp

a

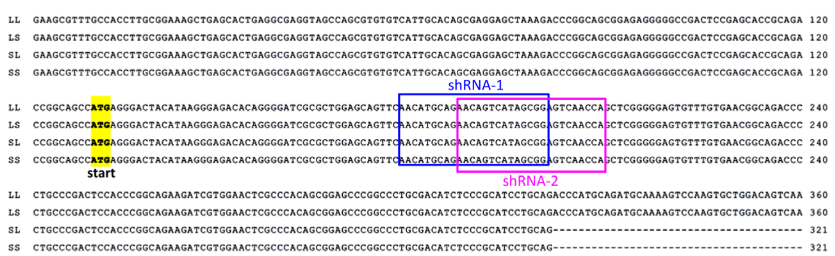

b

Control shRNA

	Fluorescence intensity		
	Weak (n=6)	Moderate (n=8)	Strong (n=8)
Undetected	4 (100%)	6 (100%)	7 (100%)
Small Eye	0	0	0
Eyeless	0	0	0
Headless	0	0	0
Abnormal	2	2	1

Pax6 shRNA-1

	Fluorescence intensity		
	Weak (n=15)	Moderate (n=11)	Strong (n=6)
Undetected	12 (100%)	6 (66.7%)	1 (25%)
Small Eye	0	3 (33.3%)	3 (75%)
Eyeless	0	0	0
Headless	0	0	0
Abnormal	3	2	2

Pax6 shRNA-2

	Fluorescence intensity		
	Weak (n=16)	Moderate (n=11)	Strong (n=7)
Undetected	9 (90%)	5 (62.5%)	0
Small Eye	1 (10%)	3 (37.5%)	1 (16.7%)
Eyeless	0	0	3 (50%)
Headless	0	0	2 (33.3%)
Abnormal	6	3	1

Supplementary Figure 1 | Results from Pax6 knock down. a, 5′ parts of cDNAs encoding isoforms (LL, LS, SL, SS) of *Cynops pyrrhogaster* Pax6 (DDBJ/GenBank Accession #: D88741). Boxes show the sequences based on which the shRNAs were designed. **b,** Effects of shRNAs on eye morphogenesis in the newt. Transgenesis was carried out and larvae showing mCherry fluorescence in the whole body were selected (see Methods). In the control shRNA, which was designed from the newt crystallin promoter (DDBJ/GenBank Accession #: AB113881), abnormalities were not recognized in larvae regardless of the fluorescence intensity of mCherry (an indicator for the relative expression level of shRNA), except for a curved tail fin, which was also observed in another control group with an empty construct (data not shown). Therefore, the animals showing such an abnormality (numbers shown in the last row of each table) were omitted from data analysis. In Pax6 shRNA-1, a small eye phenotype was observed in 33.3% and 75% of animals which showed moderate and strong fluorescence, respectively. On the other hand, effects of Pax6 shRNA-2 seemed to be much more severe: 33% and 50% of animals showing strong fluorescence exhibited headless and eyeless phenotypes, respectively (as an example of eyeless larva, see Fig. 1b). An aspect that is correlated to the severity of the cranial part should be noted, namely that the total length along the body axis tended to decrease. Such abnormalities were consistent with those reported in other vertebrates[1-3].

References

1. Hill, R. E., Favor, J., Hogan, B. L. M., Ton, C. C. T., Saunders, G. F., Hanson, I. M., Prosser, J., Jordan, T., Hastie, N. D. & van Heyningen, V. Mouse *Small eye* results from mutations in a paired-like homeobox-containing gene. *Nature* **354,** 522-525 (1991).

2. Matsuo, T., Osumi-Yamashita, N., Noji, S., Ohuchi, H., Koyama, E., Myokai, F., Matsuo, N., Taniguchi, S., Doi, H., Iseki, S., Ninomiya, Y., Fujiwara, M., Watanabe, T. & Eto, K. A mutation in the Pax-6 gene in rat *small eye* is associated with impaired migration of midbrain crest cells. *Nature Genet.* **3,** 299-304 (1993).

3. Peng, S., York, J. P. & Zhang, P. A transgenic approach for RNA interference-based genetic screening in mice. *Proc. Natn. Acad. Sci. U. S. A.* **103,** 2252-2256 (2006).

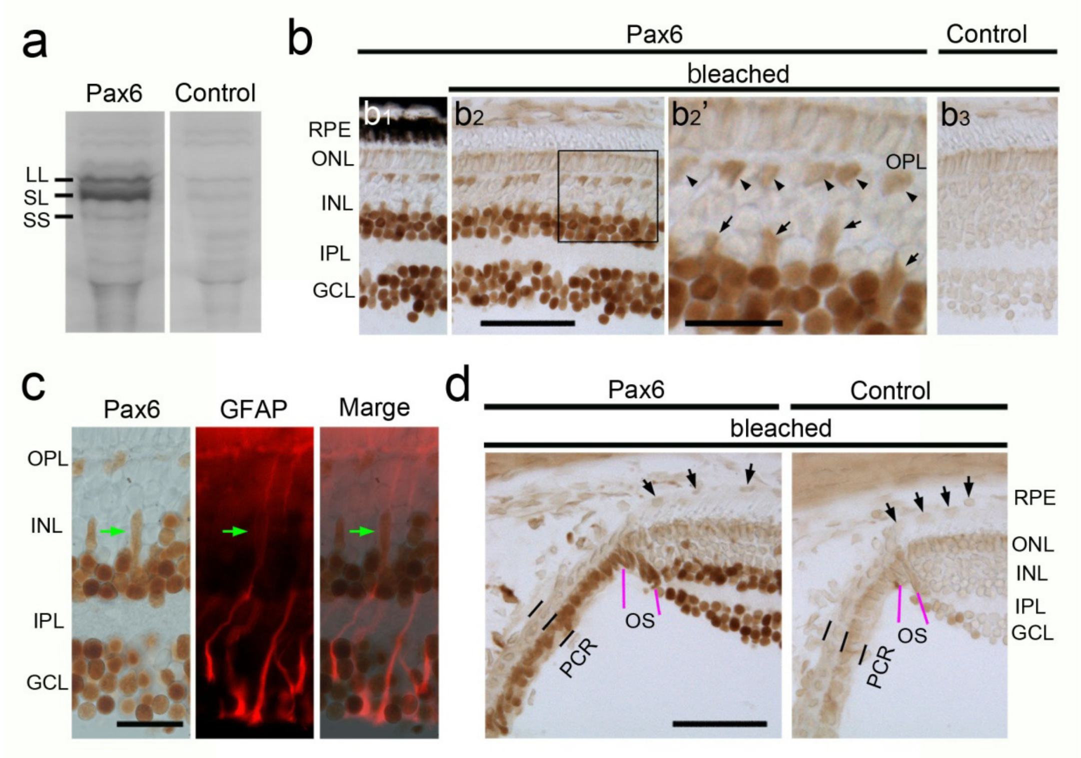

Supplementary Figure 2 | Expression pattern of Pax6 in adult normal retina. a, Western blot of the protein extracted from the neural retina. Pax6 antibody (AD2.38) labelled protein bands corresponding to the Pax6 isoforms, LL (49 kD), SL (47 kD) and SS (45 kD). **b, c,** Immunohistochemistry of the central retina. AD2.38 labelled almost all of the cell bodies (nuclei) of amacrine and ganglion cells, which are located in the inner one third of the inner nuclear layer (*INL*) and in the ganglion cell layer (*GCL*). Labelling intensity was not uniform. This antibody also labelled cell bodies (nuclei) of Müller glia cells (arrows) and horizontal cells (arrowheads) (**b_2'**). b_1: before bleaching; b_2 and b_3: after bleaching. b_2': enlargement of the box in b_2. Pax6-immunoreactivity in Müller glia cells (green arrow) were confirmed by double labelling with a GFAP antibody (red) which visualizes the apical and basal processes of Müller glia cells[1] (**c**). **d,** Immunohistochemistry of the peripheral retina (after bleaching). The ciliary marginal zone (CMZ), which is comprised of *ora serrata* (*OS*) and *pars ciliaris retinae* (*PCR*: the partially pigmented inner layer of the ciliary epithelium)[2], containing the retinal stem/progenitor cells, was labelled with AD2.38. Arrows indicate nuclei of RPE cells with no labelling. *ONL*: outer nuclear layer; *OPL*: outer plexiform layer; *IPL*: inner plexiform layer. Scale bars: 100 μm for **b_2** and **d**; 40 μm for **b_2'** and **c**.

References

1. Susaki, K. & Chiba, C. MEK mediates *in vitro* neural transdifferentiation of the adult newt retinal pigment epithelium cells: Is FGF2 an induction factor? *Pigment Cell Res.* **20,** 364-379 (2007).

2. Chiba, C., Hoshino, A., Nakamura, K., Susaki, K., Yamano, Y., Kaneko, Y., Kuwata, O., Maruo, F. & Saito, T. Visual cycle protein RPE65 persists in new retinal cells during retinal regeneration of adult newt. *J. Comp. Neurol.* **495,** 391-407 (2006).

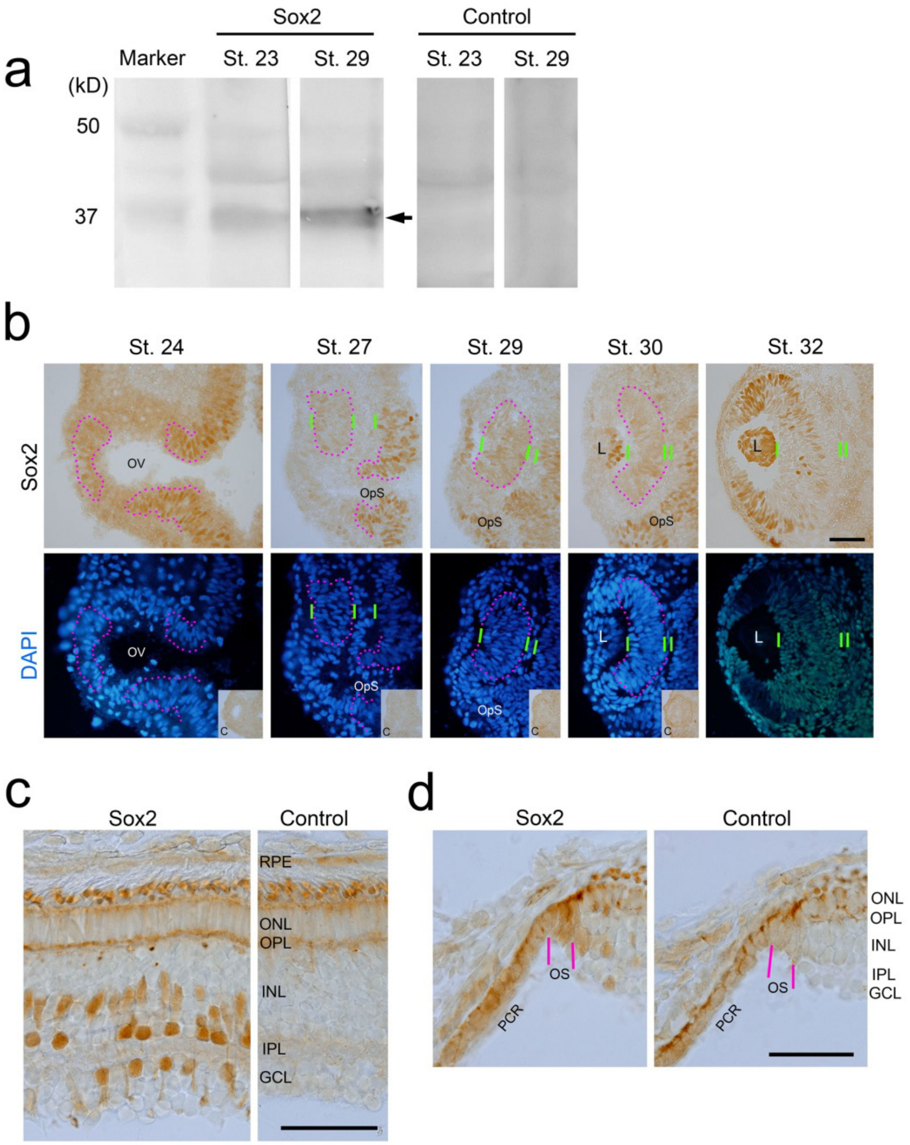

Supplementary Figure 3 | Expression patterns of Sox2 in developing and adult normal retinas. a, Western blot of the protein extracted from embryos (St. 23 and St. 29). Sox2 antibody (ab97959) labelled protein bands corresponding to the newt Sox2 (~34 kD) in both stages. **b,** Immunohistochemistry of developing retinas (after bleaching). Sox2-immunoreactivity, which was observed uniformly in nuclei of the early optic vesicle, was restricted in the pro-NR regions in the late optic vesicle (St. 24) to the optic cup (St. 29). The immunoreactivity observed in the retinal stem/progenitor cells in the central retina (St. 30) became restricted in the peripheral retina as neuronal differentiation started from the central retina while the retina grew in the periphery (St. 32). Inset panels labelled with 'c': negative control; DAPI: a nuclear marker; pink dotted lines: Sox2-immunoreactive area; short green lines: borders of NR and the RPE; *OV*: the optic vesicle; *L*: the lens; *OpS*: the optic stalk. **c, d,** Immunohistochemistry of adult normal retina (after bleaching). ab97959 labelled a small number of cell bodies (nuclei), possibly those of amacrine cells, which line both the outer and inner margins of the inner plexiform layer (*IPL*), as well as cell bodies and basal processes of Müller glia cells (**c**). In the ciliary marginal zone (CMZ), *ora serrata* (*OS*) was also labelled, but in *pars ciliaris retinae* (*PCR*) immunoreactivity was not clear. *ONL*: outer nuclear layer; *OPL*: outer plexiform layer; *IPN*: inner nuclear layer; *GCL*: ganglion cell layer. Scale bars: 100 µm.

Supplementary Table 1. Inferred expression patterns of Pax6, Mitf, c-Myc, Klf4, Sox2 and RPE65 in retinal development.

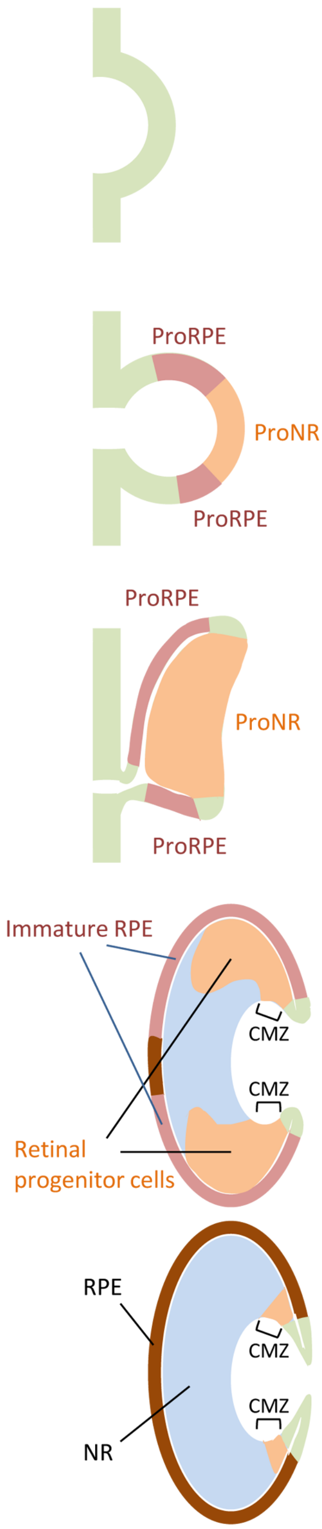

		Pax6	Mitf	c-Myc	Klf4	Sox2	RPE65
Optic vesicle (Early)		✓	[1,2,3,4] ✓	[5] ✓	[4] ✓	✓	ND
Optic vesicle (Late)	ProNR	✓	[1,2,3,4] ND	[5] ✓	[4] ✓	✓	ND
	ProRPE	✓	[1,2,3,4] ✓	[5] ✓	[4] ✓	ND	ND
Optic cup	ProNR	✓	[1,2,3,4] ND	[4,5,6] ✓	[4] ✓	✓	ND
	ProRPE	✓	[1,2,3,4] ✓	[4] ✓	[4] ✓	ND	ND
Immature retina	Retinal progenitor cells	✓	[1,2,3,4] ND	[5] ND	[4] ✓	✓	ND
	Immature RPE cells	✓	[1,2,3,4] ✓	[4] ✓	[4] ✓	ND	ND
	CMZ	✓	[1,2,3,4] ND	[4,5,6] ✓	[4] ✓	✓	ND
Mature retina	NR	✓ (G, A, H, M)	[1,2,3,4] ND	[5,6] ND	[7,8] ✓ (G)	✓ (A, M)	ND
	RPE	ND	ND	ND	ND	ND	✓
	CMZ	✓	[1,2,3,4] ND	[4,5,6] ✓	[4] ✓	✓	ND

✔: expression (determined in this study); ND: no detection (determined in this study); ✔: expression (inferred from other studies; #: reference); ND: no detection (inferred from other studies; #: reference); NR: neural retina; RPE: retinal pigment epithelium; CMZ: ciliary marginal zone; G: ganglion cells; A: amacrine cells; H: horizontal cells; M: Müller glial cells

References for Supplementary Table 1

1. Bharti, K., Liu, W., Csermely, T., Bertuzzi, S. & Arnheiter, H. Alternative promoter use in eye development: complex role and regulation of the transcription factor MITF. *Development*. **135,** 1169–1178 (2008).
2. Bora, N., Conway, S. J., Liang, H. & Smith, S. B. Transient overexpression of the *Microphthalmia* gene in the eyes of *Microphthalmia vitiligo* mutant mice. *Dev. Dyn.* **213,** 283-292 (1998).
3. Kumasaka, M., Sato, H., Sato, S., Yajima, I. & Tamamoto, H. Isolation and developmental expression of *Mitf* in *Xenopus laevis*. *Dev. Dyn.* **230,** 107-113 (2004).
4. Luz-Madrigal, A., Grajales-Esquivel, E., McCorkle, A., DiLorenzo, A. M., Barbosa-Sabanero, K., Tsonis, P. A. & Del Rio-Tsonis, K. Reprogramming of the chick retinal pigmented epithelium after retinal injury. *BMC Biol.* **12,** 28 (2014).
5. Xue, X. Y. & Harris, W. A. Using *myc* genes to search for stem cells in the ciliary margin of the *Xenopus* retina. *Dev. Neurobiol.* **72,** 475-490 (2012).
6. Yamaguchi, M., Tonou-Fujimori, N., Komori, A., Maeda, R., Nojima, Y., Li, H., Okamoto, H. & Masai, I. Histone deacetylase 1 regulates retinal neurogenesis in zebrafish by suppressing Wnt and Notch signaling pathways. *Development* **132,** 3027-3043 (2005).
7. Gupta, D., Harvey, S. A. K., Kenchegowda, D., Swamynathan, S. & Swamynathan, S. K. Regulation of mouse lens maturation and gene expression by Krüppel-like factor 4. *Exp. Eye Res.* **116,** 205-218 (2013).
8. Moore, D. L., Blackmore, M. G., Hu, Y., Kaestner, K. H., Bixby, J. L., Lemmon, V. P. & Goldberg, J. L. KLF family members regulates intrinsic axon regeneration. *Science* **326,** 298-301 (2009).
